# First evidence of established populations of the taiga tick *Ixodes persulcatus* (Acari: Ixodidae) in Sweden

**DOI:** 10.1186/s13071-016-1658-3

**Published:** 2016-07-01

**Authors:** Thomas G. T. Jaenson, Kairi Värv, Isabella Fröjdman, Anu Jääskeläinen, Kaj Rundgren, Veerle Versteirt, Agustín Estrada-Peña, Jolyon M. Medlock, Irina Golovljova

**Affiliations:** 1 Medical Entomology Unit, Department of Organismal Biology, Evolutionary Biology Centre, Uppsala University, Norbyvägen 18d, SE-752 36 Uppsala, Sweden; 2 Department of Virology, National Institute for Health Development, Hiiu 42, 11619 Tallinn, Estonia; 3 Aalto University, P.O. Box 11130, FI-00076 Aalto, Finland; 4 Department of Virology, University of Helsinki, P.O. Box 21, FI-00014 Helsinki, Finland; 5 Järvis väg 50, SE-953 95 Nikkala, Sweden; 6 Precision Pest Management Unit, Avia-GIS, Risschotlei 33, BE-2980 Zoersel, Belgium; 7 Department of Parasitology, University of Zaragoza, Miguel Servet 177, ES-50013 Zaragoza, Spain; 8 Medical Entomology Group, Emergency Response Department, Public Health England, Porton Down, Salisbury, UK; 9 Health Protection Research Unit in Emerging Infections and Zoonoses, Porton Down, Salisbury, UK

**Keywords:** *Ixodes persulcatus*, *Ixodes ricinus*, Taiga tick, Geographical distribution, Sweden, Bothnian Bay, Norrbotten, Moose, *Alces alces*, Tick-borne pathogens

## Abstract

**Background:**

The tick species *Ixodes ricinus* and *I. persulcatus* are of exceptional medical importance in the western and eastern parts, respectively, of the Palaearctic region. In Russia and Finland the range of *I. persulcatus* has recently increased. In Finland the first records of *I. persulcatus* are from 2004. The apparent expansion of its range in Finland prompted us to investigate if *I. persulcatus* also occurs in Sweden.

**Methods:**

Dog owners and hunters in the coastal areas of northern Sweden provided information about localities where ticks could be present. In May-August 2015 we used the cloth-dragging method in 36 localities potentially harbouring ticks in the Bothnian Bay area, province Norrbotten (NB) of northern Sweden. Further to the south in the provinces Västerbotten (VB) and Uppland (UP) eight localities were similarly investigated.

**Results:**

*Ixodes persulcatus* was detected in 9 of 36 field localities in the Bothnian Bay area. Nymphs, adult males and adult females (*n* = 46 ticks) of *I. persulcatus* were present mainly in *Alnus incana - Sorbus aucuparia - Picea abies - Pinus sylvestris* vegetation communities on islands in the Bothnian Bay. Some of these *I. persulcatus* populations seem to be the most northerly populations so far recorded of this species. Dog owners asserted that their dogs became tick-infested on these islands for the first time 7–8 years ago. Moose (*Alces alces*), hares (*Lepus timidus*), domestic dogs (*Canis lupus familiaris*) and ground-feeding birds are the most likely carriers dispersing *I. persulcatus* in this area. All ticks (*n* = 124) from the more southern provinces of VB and UP were identified as *I. ricinus*.

**Conclusions:**

The geographical range of the taiga tick has recently expanded into northern Sweden. Increased information about prophylactic, anti-tick measures should be directed to people living in or visiting the coastal areas and islands of the Baltic Bay.

**Electronic supplementary material:**

The online version of this article (doi:10.1186/s13071-016-1658-3) contains supplementary material, which is available to authorized users.

## Background

Two closely related tick species are of particular medical importance as vectors of zoonotic viruses, bacteria and protists in the Palaearctic region: the castor bean tick *Ixodes ricinus* in Europe and the taiga tick *I. persulcatus* in eastern Europe and northern Asia [1–3]. *Ixodes persulcatus* is the main vector of the Siberian and Far Eastern subtypes of the tick-borne encephalitis virus (TBEV) in northern Asia and eastern Europe [4, 5]. In some localities in its western range *I. persulcatus* is also a vector of the European subtype of TBEV [4, 6–8] the usual vector of which is *I. ricinus.* However, in a few localities (in Estonia, western Russia and south-eastern Finland) even *I. ricinus* may transmit the Siberian subtype [8, 9]. Several other zoonotic pathogens have been detected in *I. persulcatus*, e.g. Powassan virus [4], Kemerovo virus [2, 6], *Borrelia miyamotoi*, *B. afzelii, B. garinii*, *B. bavariensis, B. valaisiana* [10], *Anaplasma phagocytophilum* [11, 12], *Ehrlichia* spp. [13], *Candidatus Neoehrlichia mikurensis* [12], *Bartonella* spp. [14], *Rickettsia helvetica* and other *Rickettsia* species [15], *Babesia microti* [16], *Ba. venatorum* [12] and *Ba. divergens* [17]. In contrast to *I. ricinus,* which is more often found attached as nymphs than as adult females to people, it is nearly always the adult *I. persulcatus* female, which acts as the vector of pathogens to humans [2, 18].

The geographical distribution of *I. persulcatus* ranges from a scattered distribution in Finland, to a more continuous distribution from eastern Latvia and eastern Estonia through the taiga zone of European and Asian Russia into Mongolia and the Peoples Republic of China, Taiwan and North Korea eastwards to Hokkaido and Honshu in Japan [8, 18–21]. *Ixodes persulcatus* usually inhabits mixed deciduous-coniferous forest [19, 22].

In several regions of Russia *I. persulcatus* has recently increased its range and abundance, in particular towards the north [23–26]. In Finland, *I. persulcatus* was first recorded in the Kokkola archipelago on the west coast of Finland in 2004 [27] and in Närpiö (Närpes) in 2008 [28]. In the Kokkola archipelago *I. persulcatus* transmits the Siberian subtype of the TBE virus [27]. In 2008 and 2009 four human TBE cases were reported further to the north, i.e. from Simo (65°40′N, 24°54′E) in Finnish Lapland [29]. Remarkably, the TBEV vector at Simo is *I. persulcatus* but here it transmits the European subtype of TBEV [29]. Thereafter, *I. persulcatus* was collected in Muhos in north-central Finland in May 2015 (Anu Jääskeläinen, unpublished observations) and in eastern Finland in Mekrijärvi and Kuhmo in 2008 and 2009 [30]. Based on ticks sent by the general public to the University of Turku (Åbo) in 2015, *I. persulcatus* seems now to have a wider distribution in Finland [31]. Immature ticks originating from the *I. persulcatus* populations in the Baltic region, Finland and European Russia could act as sources for the northward and northwestward spread of *I. persulcatus* by e.g. spring-migrating birds into Sweden. Even birds and mammals moving westwards might disperse ticks from northern Russia and Finland into Sweden.

The aim of this investigation was to determine if *I. persulcatus* is present in northern Sweden (provinces of Norrbotten and Västerbotten) and if this tick species is also present further to the south in eastern Sweden (in the province of Uppland). This decision was based on the following facts: (i) it has been estimated that ≈ 7 million ticks are carried into Sweden each spring by migratory birds [32]; (ii) many of these ticks could originate from the Baltic countries and Finland, where *I. persulcatus* is present [7, 8, 21]; (iii) in 1992 we found a nymph of *I. persulcatus* on a warbler captured on a small island near the coast of northern Sweden [33]; (iv) *I. persulcatus* has recently expanded its range in northern European Russia [23, 24] and Finland; (v) and because climate change is likely to increase the possibility for *I. persulcatus* to become established in northern Sweden [26, 34].

## Methods

### Study areas and climate

**Fig. 1:**
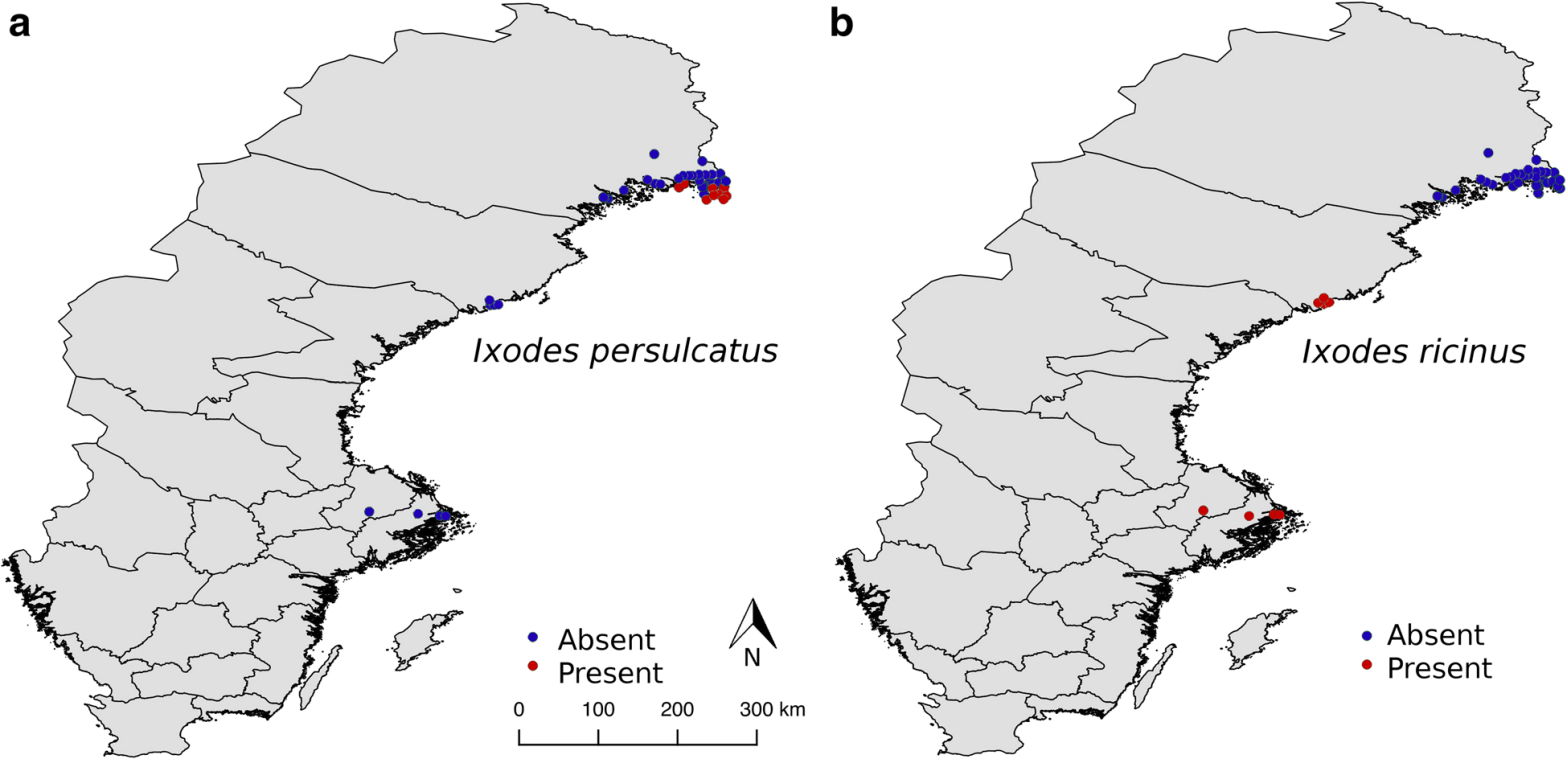
**a** Map of Sweden showing localities where *Ixodes persulcatus* (*filled red circles*) was collected in 2015. Filled blue circles indicate localities where *I. persulcatus* was not recorded; **b** Map of Sweden showing localities where the tick *I. ricinus* (*filled red circles*) was collected in 2015. Filled blue circles indicate localities where *I. ricinus* was not recorded

The main study locations are situated in the provinces of Norrbotten (NB) and Västerbotten (VB) in northern Sweden (Fig. 1). These provinces are to the east bordered by the the Bothnian Bay and the Gulf of Bothnia, respectively and belong to the middle boreal subzone [35]. Norrbotten is to the west bordered by the Swedish province Lappland, which together with Norrbotten form the northernmost provinces of Sweden. This is a typical taiga region, strongly dominated by forests but also in part by mires. It reaches in altitude to about 300 m a.s.l. in Västerbotten and to 150–200 m a.s.l. in Norrbotten. In the Bothnian Bay area, where we found *I. persulcatus* at several localities, the length of the vegetation period is about 140–150 days and the annual precipitation 600–700 mm [35]. The July mean temperature is 15–16 °C [35]. This district is characterised by many large and small water-courses, lakes, extensive pine and spruce forests, coasts formed from isostatic, continuous uplift of the landmass after the last Ice age, brackish sea water and > 6,000 islands. The human population density is low compared with southern Sweden. Most inhabitants of the Bothnian Bay area reside in the coastal regions and the river valleys [36].

About 280 km to the southwest of the Bothnian Bay area, four localities in the Norrbyskär archipelago, VB were investigated. Here, the length of the vegetation period is on average 150–160 days and the annual mean precipitation 700–800 mm [35]. Further to the south in the Swedish province Uppland (UP) located in the boreonemoral zone, four localities were investigated (Additional file 1: Table S1). Here, the mean duration of the growing season is 180–190 days [35].

### Tick collection localities

We contacted dog owners and hunters living in the coastal areas of the province of Norrbotten of northern Sweden and asked if they and/or their dogs had become infested by ticks in the Baltic Bay area. We then asked for the name(s) of the presumed locality/localities where any tick infestation had occurred. Based on this information we searched for ticks at a total of 36 localities in the Bothnian Bay area, NB during early May and in July and August 2015.

In addition, we searched for ticks further to the south in Sweden, in the provinces of Västerbotten (four localities) and Uppland (four localities) during August - October 2015. More information about each sampling locality is provided in Additional file 1: Table S1.

### Tick collection

Sampling for ticks by the cloth-dragging method took place once at each of 44 localities during May - October 2015 (Additional file 1: Table S1). All sampling sessions took place during daytime when the ground vegetation was not wet. A woollen 1 m^2^ cloth was pulled at slow walking pace over the ground vegetation and/or field vegetation for a total of 300 m. At every 10 m of dragging both sides of the cloth were inspected with the help of a magnifying lens. Any tick detected was put in a numbered plastic vial containing 80 % ethanol. If no tick had been collected after 30 stops (300 m^2^ of blanket-dragging) another dragging-round of 30 stops (300 m^2^) was performed. At each sampling occasion the following parameters were recorded: time of the day, weather conditions, composition of the plant community and observation of mammal species and signs of their presence, temperature and relative humidity measured at ground level before and after each sampling period, and the location (longitude and latitude) of each sampling site (Additional file 1: Table S1).

### Tick identification

All tick specimens were identified to stage and species by morphological characteristics. A Leitz Wild M10 stereomicroscope with possibility of magnifications up to × 200 was used together with keys and illustrations in Filippova [3] and Yamaguti et al. [19]. By these means the unfed nymphs, adult females and adult males (Additional file 3: Figure S1) of *I. persulcatus* could be distinguished from the closely related *I. ricinus.* To further confirm the species diagnoses the morphology of the ticks collected in the Baltic Bay area was compared with that of ethanol-preserved *I. persulcatus* nymphs and adult male and female ticks collected in several Russian regions (St. Petersburg, Permsk, Khabarovsk, Sakhalin). The key (Additional file 2: Table S2) provides morphological characteristics, partly compiled from [3, 19], which were used to distinguish *I. persulcatus* from *I. ricinus*.

### Molecular tick species identification

In order to check the species diagnoses based on morphological characteristics (Additional file 2: Table S2) we performed molecular species identification (see below) of 10 specimens morphologically identified as “*I. persulcatus”* from Norrbotten (9 females, 1 nymph) and 11 specimens morphologically identified as “*I. ricinus*” from Västerbotten (4 females, 3 nymphs) or Uppland (1 female, 3 nymphs).

Collected ticks were homogenized individually with Mixer Mill MM301 bead-milling system (Retsch GmbH, Haan, Germany) in 300 μl of sterile phosphate-buffered saline (PBS). Nucleic acid extraction was performed with guanidinium-phenol-chloroform method (TriPure Isolation system, Roche Diagnostics) according to manufacturer’s instructions from 200 μl of tick suspension. Sterile water was included as a negative control in every preparation set. The extracted DNA and RNA were stored at -20 °C and -70 °C, respectively, prior to use.

Mitochondrial partial 16S rRNA gene was used as a marker for the identification of tick species using primers 16Sa and 16Sb, as previously described [37]. The reaction was performed in a total volume of 25 μl, and the reaction mixture included 10× PCR buffer (Dream Taq, Thermo Scientific, Waltham, USA), per 1.5 mM of MgCl_2_ and 1.5 mM dNTPs (Thermo Scientific, Walthan, USA), 0.5 μM of each primer and 2.5 U of DreamTaq polymerase (Thermo Scientific, Waltham, USA). Cycling conditions were as follows: 95 °C for 1 min; 49 °C for 1 min; 72 °C for 2 min; 95 °C for 1 min; 47 °C for 1 min; 72 °C for 2 min; followed by 40 cycles of 95 °C for 30 s, 45 °C for 1 min and 72 °C for 2 min; and a final elongation step of 72 °C for 10 min. PCR reactions were carried out on a ThermoCycler 2720 (Applied Biosystems, Thermo Fisher Scientific, Waltham, USA). All PCR products were sent for direct sequencing at the Estonian Biocentre (Tartu, Estonia). Obtained sequences were aligned and analyzed with BioEdit v. 7.0.9.0 software.

## Results

### Tick species in Norrbotten

A total of 46 *I. persulcatus* ticks (13 nymphs, 20 adult females and 13 adult males) was found in July and August at eight insular and one coastal locality in the Bothnian Bay area (Fig. 1a, Table 1; Additional file 1: Table S1). *Ixodes ricinus* was not found at any of the 36 localities surveyed in this area (Additional file 1: Table S1). This is the only area in Sweden where we have, so far, detected populations of *I. persulcatus*. The dominant plant community in which *I. persulcatus* was found can be classified as virgin mixed forest dominated by grey alder (*Alnus incana*), rowan (*Sorbus aucuparia*), Norway spruce (*Picea abies*) and Scots pine (*Pinus sylvestris*) (Additional file 4: Figure S2). Dog owners living at localities where we found *I. persulcatus*, informed us that “ticks began to infest their dogs 7–8 years ago”. This suggests that the taiga tick became established in this area about ten years ago. Moreover, we received additional information of tick presence from people that they and/or their dogs had become infested by ticks on the following islands: Seskarö, Haparanda Sandskär, Västra Knivskär, Östra Knivskär, Stora Hamnskär, Hanhinkari and Ytterstlandet.

**Table 1:** Localities in the province of Norrbotten, North Sweden where Ixodes persulcatus* was collected by cloth-dragging during July–August 2015. Additional information is available in Additional file 1: Table S1

Locality number, name and vegetation type	Potential main tick reproduction tick	Date of tick collection in 2015	No. of nymphs	No. of females	No. of males	Total no. of ticks recorded on 600 m^2^
Västra Knivskär	moose^a^, hare^b^	14 July	7	15	10	32
Virgin mixed forest						
Ytterstlandet						
Virgin mixed forest	moose^a^, hare^b^	6 August	0	1	1	2
Ytterstlandet						
*Juniperus* heath	moose^a^, hare^b^	6 August	3	0	0	3
Östra Knivskär						
Virgin mixed forest	moose^a^, hare^b^	9 August	0	1	0	1
Östra Knivskär						
Virgin mixed forest	moose^a^, hare^b^	9 August	1	0	0	1
Östra Knivskär						
Virgin mixed forest	moose^a^, hare^b^	9 August	2	0	0	2
Stora Hamnskär						
Virgin mixed forest	moose^a^, hare^b^, dog^c^	9 August	0	1	1	2**
Stora Hamnskär						
Virgin mixed forest	moose^a^, hare^b^, dog^c^	9 August	0	0	1	1
Axelsvik						
Virgin mixed forest	moose^a^ hare^b^, roe deer^d^ reindeer^e^	11 August	0	2	0	2

*All specimens were identified as *I. persulcatus* by morphological features (Additional file 2: Table S2)

Ten specimens were also identified as *I. persulcatus* by molecular criteria

**Two specimens were collected from a dog resident on Stora Hamnskär

^a^ *Alces alces*; ^b^ *Lepus timidus*; ^c^ *Canis lupus familiaris*; ^d^ *Capreolus capreolus*; ^e^ *Rangifer tarandus*

### Tick species in Västerbotten

Four separate islands in the Norrbyskär archipelago were surveyed for ticks in August 2015. All 78 specimens were identified morphologically as *I. ricinus. Ixodes persulcatus* was not found among ticks collected at Norrbyskär.

### Tick species in Uppland

A total of 46 tick specimens, all identified morphologically as *I. ricinus*, was collected from the four sampling sites in Uppland. *Ixodes persulcatus* was not found among ticks collected in Uppland.

### Molecular diagnosis of tick species

The species identity of ten tick specimens morphologically identified as *I. persulcatus* from Norrbotten (9 females, 1 nymph) and 11 specimens morphologically identified as *I. ricinus* from Västerbotten (4 females, 3 nymphs) or Uppland (1 female, 3 nymphs) was verified by 16S rRNA sequencing. In all cases molecular identification correlated positively with morphological species discrimination. The intraspecies similarity between obtained 16S rRNA sequences was on average 98.7 % and 99.9 % for *I. ricinus* and *I. persulcatus*, respectively. Nine *I. persulcatus* ticks out of ten had identical 16S rRNA sequences; the remaining sequence differed by one nucleotide deletion at position 304. *Ixodes ricinus* sequences demonstrated higher variability with similarity values ranging from 97.4 % to 100 % between samples. 16S rRNA sequences from Swedish *I. ricinus* and *I. persulcatus* ticks were aligned and compared to complete and partial 16S rRNA sequences available in the NCBI DNA database. Swedish *I. ricinus* sequences were highly similar or identical with previously published sequences from Germany (JF928527), France (GU074616), Slovakia (GU074590), Ireland (GU074595), Estonia (GU074594), and Lithuania (KT070766, KT070770, KT070778). Obtained *I. persulcatus* sequences were 99.6–100 % identical with the following GenBank submissions: Japan, Aomori (AB819250), Russia (JF934741), Russia, East Siberia (KP866204) and Lithuania (KP283020).

## Discussion

### The geographical expansion of *Ixodes persulcatus*

Here we report the presence of *I. persulcatus* in the Province of Norrbotten, Bothnian Bay area, northern Sweden. Before this study there was only one previous record of *I. persulcatus* from Sweden: one nymph was removed from a willow warbler *Phylloscopus trochilus* captured on 19 May 1992 on the island of Stora Fjäderägg in the Bothnian Sea [33] about 100 km to the west of the Kokkola archipelago, Finland where *I. persulcatus* was discovered in 2004 [27].

Investigations show that the reproductive ranges of *I. ricinus* and *I. persulcatus* in northern Europe seem to have increased towards the north during the last few decades. This applies to *I. ricinus* in Norway [38, 39] and Sweden [40]. In Sweden *I. ricinus* has increased its abundance and geographical distribution northwards during the last two decades [33]. This tick species is now abundant in southern and south-central Sweden and along the Baltic Sea coast to about 64 °N. North of this latitude there have been many scattered records of *I. ricinus* since the 1980s [33].

In north-western European Russia *I. persulcatus* has extended its range and has also become more abundant [23, 24]. In Karelia, Bugmyrin and co-workers recorded a significantly increased abundance of *I. persulcatus* and a decrease in *I. ricinus* abundance since the 1950s [23]. Southern Karelia is presently a sympatric zone for *I. ricinus* and *I. persulcatus* [23]. In most of Karelia, except to the west of Lake Ladoga, *I. persulcatus* is now the most abundant of the two tick species [23]. In the Archangelsk region, Tokarevich and co-workers recorded an increased abundance of *I. persulcatus* as well as a drastically increased incidence of human TBE [24]. This increased TBE incidence is considered to be related to the increasing mean annual air temperature in that area [24]. Since *I. persulcatus* has recently extended its reproductive range towards the north and north-west in north-western Russia and in Finland, we may expect that the range of this tick species will soon become larger even in Sweden.

### Climatic requirements of *I. persulcatus* seem to differ from those of *I. ricinus*

In the Bothnian Bay area, where we found *I. persulcatus* at several localities, and where permanent populations of *I. ricinus* appear not to exist, the mean length of the vegetation period is 140–150 days [35]. We have previously shown that *I. ricinus* is commonly encountered in areas where the vegetation period is > 180 days/year and is absent from areas with a vegetation period *<* 160 days/year [41]. An annual vegetation period of 170 days was found to have a good fit with the northern distribution limit of *I. ricinus* [41]. Another climate index, which like the vegetation period is dependent on the latitude and altitude above sea level, is the temperature sum (T_S_). This is the total daily mean temperature above +5 °C during the vegetation period and is an index of how warm this season is. In the Bothnian Bay area T_S_ = 900–1,100 day-degrees, at Norrbyskär T_S_ = 1,100–1,300 day-degrees, and in the province of Uppland T_S_ =1,300–1,500 day-degrees [42]. Total mean precipitation during the growing season is for these three areas 250–300 mm, 250–300 mm and 300–450 mm, respectively [42]. Another potentially relevant climate index is the humidity during the growing season. This climate index could be relevant in the study of small organisms like *Ixodes* ticks, which are sensitive to a high saturation deficit (~ low humidity). In relation to other parts of Sweden the summers in eastern Sweden are, in general, relatively dry with little rainfall. And in fact, the Baltic Bay area, the Norrbyskär archipelago, and the province of Uppland have an almost identical and quite low humidity index, i.e. minus 50–0 during the vegetation period [42]. In view of the present findings of *I. persulcatus* in the Bothnian Bay region and the climatological data presented here we believe that *I. persulcatus* is able to live and reproduce in a region of North Sweden where the mean annual vegetation period duration is relatively very short or approximately 140–150 days. We suspect that the length of this period is here too short for *I. ricinus*. The reason may be that the temperature sum is too low. In other words, the summers in this northerly area are too cold and do not provide sufficient energy to permit permanent reproducing populations of *I. ricinus* to survive in the Baltic Bay area.

### What is the origin of the *I. persulcatus* ticks in the Baltic Bay area?

The main hosts of the adult *I. persulcatus* ticks are wild ungulates, particularly roe deer (*Capreolus capreolus*), red deer (*Cervus elaphus*), European moose (*Alces alces*), reindeer (*Rangifer tarandus*) and hares (*Lepus* spp.) [18, 19, 22]. The nymphs feed on many species of ground-feeding birds, small mammals and hares, squirrels and hedgehogs. The larvae rarely attack large mammals but feed mainly on small mammals and ground-feeding birds [18, 22]. As moose and the varying hare (*L. timidus*) are the most abundant medium-sized to large mammals on the islands where we found *I. persulcatus* it is likely that these mammals are the most important maintenance hosts (reproduction host) for this tick species in this area. It is presumably also the moose, the varying hare and domestic dog together with ground-feeding birds, which disseminate *I. persulcatus* to previously uninfested islands and coastal localities.

Reindeer (*Rangifer tarandus*) is another abundant, potential tick-disseminator in this area. Like moose and hares, reindeer is an excellent swimmer and can, in summer and early autumn swim between islands in the Baltic Bay archipelago. However, to control infestations by larvae of the reindeer warble fly (*Oedemagena tarandi*) and the reindeer throat botfly (*Cephenemyia trompe*), most of these semi-domesticated cervids are treated once a year with ivermectin or doramectin [43]. These drugs are effective for controlling the myiasis-causing oestrid flies mentioned, but are also used against parasitic nematodes and ectoparasitic arthropods, e.g. mites and ticks [43]. This may imply that in areas of northern Fennoscandia where reindeer are usually treated with any of these substances, such reindeer would presumably have a negative impact on the fecundity and potential vectorial capacity of *I. persulcatus.*

The populations of *I. persulcatus* now established on islands in the Baltic Bay area most likely originated from populations that have recently been detected on islands and along the coast of western and north-western Finland. Ground-feeding migratory birds are important for both short and long distance distribution of ticks. Moose, reindeer, hares, domestic dogs and other mammals could also be responsible for the spread of *I. persulcatus*. In contrast to most birds, large and medium-sized mammals are often infested by adult ticks. Such mammals, rather than small birds, could therefore be a more effective way of transporting potentially reproducing ticks, which could become effective founders of new *I. persulcatus* populations.

### Transmission of pathogens by *I. persulcatus*

*Ixodes persulcatus* is one of the most important vectors of human-pathogenic viruses and microorganisms in the temperate area of the Palaearctic region. Some of the pathogens vectored by this tick species may cause serious disease and even death in humans. Prior to the discovery of *I. persulcatus* in Finland *I. ricinus* was the only known vector of TBEV in Finland*.* Most human TBE cases were reported from the Åland archipelago in the Baltic Sea. These TBE cases were all considered to have been caused by the western subtype of the TBE virus [44]. In view of the TBEV focus at Simo [29] in northwestern Finland, less than 100 km from some of the Swedish islands infested by *I. persulcatus*, it is likely that the TBE virus is already present or will soon be present in the Swedish *I. persulcatus* populations. Introduction of the TBE virus to new localities where a potential vector exists may occur in several ways, such as: (i) with infected birds; (ii) with infected small or medium-sized mammals; (iii) with infected ticks attached to migratory birds; (iv) and/or with infected ticks attached to migrating cervids such as a moose and roe deer. All factors required for the establishment of one or more TBEV foci seem to be present in the Bothnian Bay area. A competent vector, *I. persulcatus*, is present in several localities. In most or all localities in the Baltic Bay area infested by *I. persulcatus*, moose is abundant and thus available as feeding and mating sites for the adult ticks. Several species of small and medium-sized mammals are competent TBEV transmission hosts [45]. Among such species present in the Bothnian Bay area are: *Myodes glareolus*, *Microtus agrestis, Sciurus vulgaris* and *Sorex araneus* [46]. One or more of these small mammal species are also proven competent reservoirs and transmission hosts for *Borrelia afzelii* [10], *B. miyamotoi* [47] and other pathogens vectored by *I. persulcatus*. Thus, if *I. persulcatus* extends its range in Sweden and becomes more abundant there, human diseases caused by the different subtypes of TBEV, Lyme borrelioses due to *B. afzelii*, *B. garinii* and other *B. burgdorferi*(*sensu lato*) genospecies, relapsing fever caused by *B. miyamotoi*, and certain rickettsioses and babesioses may become more common in people living in northern Sweden.

## Conclusions

This study showed that the geographical range of the taiga tick *I. persulcatus* has recently expanded into northern Sweden. In view of the extraordinary importance of this tick species as a vector of several pathogens of humans, domesticated mammals and livestock information via mass media and by other means about prophylactic, anti-tick measures should be directed to people living in or visiting the coastal areas and islands of the Baltic Bay. Surveillance to detect the potential presence of the taiga tick elsewhere in Sweden has been initiated.

## Abbreviations

a.s.l., above sea-level; NB, Province of Norrbotten, Sweden; NCBI, National Center for Biotechnology Information; PBS, phosphate-buffered saline; TBEV, tick-borne encephalitis virus; T_S:_ temperature sum; UP, Province of Uppland, Sweden; VB, Province of Västerbotten, Sweden

## Additional files

Additional file 1: Table S1. Description of the localities investigated for possible occurrence of *Ixodes* ticks. (DOCX 68 kb)
Additionla file 2: Table S2. Morphological characteristics distinguishing *I. persulcatus* from *I. ricinus* based on [3, 19]. (DOCX 22 kb)
Additional file 3: Figure S1.
*Ixodes persulcatus* collected on islands in the Bothnian Bay, northern Sweden in July 2015. Top row from left to right: dorsal view of nymph, adult female and adult male. Bottom row from left to right: ventral view of nymph, adult female and adult male. (TIF 32566 kb)
Additional file 4: Figure S2. Biotopes inhabited by the taiga tick, *Ixodes persulcatus* and moose, *Alces alces* in the Bothnian Bay archipelago, northern Sweden a. Mixed birch - spruce - rowan - willow and blueberry vegetation community on the island of Östra Knivskär, Haparanda municipality, 9th August 2015. b. Mixed birch - willow - grey alder woodland with field layer of bracken. Östra Knivskär, Haparanda municipality, 9th August 2015. c. Land upheaval mixed forest of grey alder, spruce, rowan, willow and Scots pine. Stora Hamnskär, Haparanda municipality, 9th August 2015. d. Mixed birch, spruce, rowan, pine and grey alder vegetation. Axelsvik, Kalix municipality, 11th August 2015. (TIF 38889 kb)
