# Supplementary material for: First evidence of established populations of the taiga tick Ixodes persulcatus (Acari: Ixodidae) in Sweden
Source: Parasit Vectors. 2016 Jul 1;9:377. doi: 10.1186/s13071-016-1658-3 (PMC5116163; doi:10.1186/s13071-016-1658-3)
Supplement: Additional file 1: Table S1. — Description of the localities investigated for possible occurrence of Ixodes ticks. (DOCX 68 kb) [file 13071_2016_1658_MOESM1_ESM.docx]

**Additional file 1: Table S1** Description of the localities investigated for possible occurrence of *Ixodes* ticks

| **Date 2015** | **Number of ticks per 300 m^2^ or 600 m^2a^** | **Name of province (landskap) and locality^b^** | **Location** | **Vegetation type (plant community) and principal potential tick reproduction hosts^c^** |
| --- | --- | --- | --- | --- |
| 7 May | 0 (TJ) | NB, Västra Nikkala | 65°48' 36"N, 23°50'58"E | Mixed Norway spruce (*Picea abies*), Scots pine (*Pinus sylvestris*), birch (*Betula*) forest. MO, RO, RE |
| 8 May | 0 (TJ) | NB, Seskarö, Ängsvägen | 65°44'06"N, 23°46'27"E | Scots pine (*Pinus sylvestris*) forest; HO, D, M, RO, HA |
| 8 May | 0 (TJ) | NB, Seskarö, Ängsvägen | 65°44'11"N, 23°46'17"E | Wet meadow (grassland); HO, DO, MO, RO, RE, HA |
| 8 May | 0 (TJ) | NB, Seskarö, Ängsvägen | 65°44'07"N, 23°46’ 17"E | Wet meadow (grassland); HO, DO, MO, RO, RE, HA |
| 10 July | 0 (TJ) | NB, Seskarö, Tjärdalsv. | 65°44'11"N, 23°46'17"E | Meadow (grassland); HO, DO, MO, RO, RE, HA |
| 11 July | 0 (TJ) | NB, Haparanda Boat Harbour | 65°46'13"N, 23°54'40"E | Roadside vegetation, abandoned field vegetation: *Epilobium, Populus tremula, Betula pubescens, Rubus idaeu*; DO, HA |
| 12 July | 0 (TJ) | NB, Seskarö near Tromsöv. crossing | 65°43'16"N, 23°45' 01"E | Grassland, cultivated for game fodder (Swedish: viltvall); MO, RE, RO, HA |
| 13 July | 0 (TJ) | NB, Haparanda Sandskär | 65°34'23"N, 23°45'32"E | Aspen (*Populus tremula*) forest – Old stand of aspen trees; MO, HA |
| 13 July | 0 (TJ) | NB, Haparanda Sandskär | 65°35'06"N, 23°44'51"E | *Juniperus* heath; MO, HA |
| 14 July | 7 nn, 10 mm and 15 ff/600 m^2^ (KR and TJ); *Ip* | NB, Västra Knivskär | 65°4'33"N, 24°6'46"E | Dense, mixed broad-leaved-spruce-pine tall herb virgin forest; MO, HA, DO |
| 15 July | 0 (TJ) | NB, Seskarö Boat Harbour | 65°44'31"N,  23°44'34"E | Roadside vegetation –lacustrine vegetation; HA, MO, DO |
| 4 Aug | 0 (TJ) | NB, South-East of Luleå, Halsön | 65°30'20"N, 21°59'58"E | Alder (*Alnus incana*) spruce-pine forest; MO, HA, DO |
| 4 Aug | 0 (TJ and IF) | NB, South-East of Luleå, Halsön | 65°30'20"N, 21°59'57"E | Grass lawns and alder-spruce-pine forest; MO, HA, DO |
| 4 Aug | 0 (TJ and IF) | NB, South-East of Luleå, Äggskär/Granön | 65°31'11"N, 21°54'47"E | Grass lawns and alder-spruce-pine forest; MO, HA, DO |
| 4 Aug | 0 (TJ and IF) | NB, South-East of Luleå, Äggskär/Granön | 65°31'02"N, 21°55'58"E | Alder-spruce-pine forest; MO; HA, DO |
| 4 Aug | 0 (TJ and IF) | NB, South-East of Luleå, Måttsund | 65°31'46"N, 21°54'44"E | Moist birch, rowan woodland near road; DO, MO, HA, RO |
| 4 Aug | 0 (TJ and IF) | NB, South-East of Luleå, Måttsund | 65°31'44"N, 21°54'56"E | Abandoned field vegetation on moist slope, *Epilobium angustifolium*; *Urtica* etc.; DO, MO, HA, RO |
| 6 Aug | 1 m, 1 f/600 m^2^ (TJ); *Ip* | NB, East of Kalix Nyborg, Ytterstlandet | 65°42'39"N, 23°17'44"E | Humid, virgin mixed alder-spruce-pine forest; MO, HA, RO |
| 6 Aug | 3nn/600 m^2^ (TJ and IF); *Ip* | NB, East of Kalix Nyborg, Ytterstlandet | 65°42'34"N, 23°17'20"E | Meadow/Lawn; MO, HA, RO |
| 7 Aug | 0 (TJ) | NB, Kukkolaforsen | 65°57'35"N, 24°02'36"E | *Picea*, tall herb community close to Torne River; MO, RE, HA, RO |
| 7 Aug | 0 (TJ) | NB, North of Haparanda, Bäckesta | 66°11'19"N, 23°42'56"E | Aspen, rowan (*Sorbus aucuparia*) , *Juniperus, Vaccinium*; MO, RE, HA, RO, DO |
| 8 Aug | 0 (TJ) | NB, North-Western part of Haparanda | 65°50'44"N,  24°07'28"E | Abandoned field vegetation and roadside vegetation, both without trees; DO |
| 8 Aug | 0 (TJ & IF) | NB, Salmis, Kirveskari | 65°47'38"N, 24°00'44"E | Mixed broad-leaved-alder, birch spruce forest; MO, RE, HA, RO, DO |
| 9 Aug | 1 f/600 m^2^ (TJ); *Ip* | NB, Östra Knivskär | 65°40'2"N, 24°8'15"E | Mixed birch-pine-rowan-spruce-willow forest; MO, HA |
| 9 Aug | 1 n/600 m^2^ (IF); *Ip* | NB, Östra Knivskär | 65°40'2"N, 24°8'15"E | Mixed birch-pine-rowan forest; MO, HA |
| 9 Aug | 2 nn/600 m^2^ (KR); *Ip* | NB, Northern part of Östra Knivskär | 65°40'3"N, 24°8'59"E | Mixed birch-pine-rowan forest; MO, HA |
| 9 Aug | 1 m, 1 f on dog (EEP); *Ip* | NB, Stora Hamnskär | 65°42'22"N, 24°06'28"E | Mixed birch-pine-rowan, forest; MO, HA, DO |
| 9 Aug | 1 m/600 m^2^ (TJ); *Ip* | NB, Stora Hamnskär | 65°42'22"N, 24°06'36"E | *Picea-Vaccinium myrtillus* low herb community; MO, HA, DO |
| 10 Aug | 0/600 m^2^ (TJ) | NB, Haparanda, Sundholmen | 65°49'06"N, 24°08'44"E | *Betula* floodplain; DO |
| 11 Aug | 2 ff /600 m^2^ (TJ); *Ip* | NB, Axelsvik | 65°46'31"N, 23°23'11"E | Dense mixed birch-spruce forest close to the Bothnian Gulf; MO, RE, HA, RO, DO |
| 12 Aug | 0/600 m^2^ (TJ and IF) | NB, Southern Seskarö, Kenkälahti | 65°42'20"N,  23°43'30"E | Mixed spruce-birch -willow forest; RE, MO, HA |
| 12 Aug | 0/600 m^2^ (TJ and IF) | NB, Southern Seskarö, Kenkälahti | 65°42'20"N,  23°43'30"E | Mainly *Pinus-Picea* tall herb virgin forest community; RE, MO, HA |
| 12 Aug | 0/600 m^2^ (TJ and IF) | NB, Southern Seskarö, Kenkälahti | 65°42'20"N,  23°43'30"E | Mainly *Picea*-tall herb forest community; RE, MO, HA |
| 13 Aug | 0/600 m^2^ (TJ and IF) | NB, Southwestern Seskarö, Järveniemi | 65°42'27"N,  23°42'10"E | Mixed broad-leaved (*Alnus incana*, *Betula, Quercus*), spruce forest community; RE, MO, HA |
| 13 Aug | 0/600 m^2^ (TJ and IF) | NB, Southwestern Seskarö, Järveniemi | 65°42'27"N,  23°42'10"E | Mixed broad-leaved (*Alnus incana*, *Betula, Quercus*), spruce forest; RE, MO, HA |
| 14 Aug | 0/600 m^2^ (TJ) | NB, Överkalix at Hansavan | 66°19'3"N, 22°50'21"E | Mixed birch, rowan, pine, willow, meadowsweet (*Filipendula ulmaria*) near lake; DO, RE, MO |
| 1. Aug | 8 nn, 37 mm and ff/600 m^2^ (TJ). *Ir* | VB, Norrbyskär, Blågrundet | 63°33'11"N, 19°52'59"E | Mixed vegetation: *Salix, Betula, Pinus, Alnus incana, Sorbus, Urtica, Rubus, Vaccinium*; DO, HA |
| 20 Aug | 8 nn, 7ff, 5 mm/600 m^2^ (TJ); *Ir* | VB, Norrbyskär, Stengrundet | 63°33'02"N, 19°52'56"E | Mixed vegetation: *Salix, Betula, Pinus, Alnus incana, Sorbus, Urtica, Rubus, Vaccinium*; DO, HA |
| 20 Aug | 1f, 2 mm, 2 nn/600 m^2^ (TJ); *Ir* | VB, Norrbyskär, Stuguskär | 63°33'24"N, 19°52'14"E | Clear-felled area with *Vaccinium myrtillus* and *Rubus*; DO, HA |
| 15-23 Aug | 5 ff, 3 mm from dog (MB). *Ir* | VB, Norrbyskär, Persskär | 63°34'01"N, 19°52'02"E | Mixed vegetation: *Salix, Betula, Pinus, Alnus incana, Sorbus, Urtica, Rubus, Vaccinium*; DO, HA |
| 30 Aug | 6 nn, 2 mm, 3ff /300 m^2^ (IF); *Ir* | UP, Rådmansö, Åkerögården | 59°44'05"N, 18°56'39"E | Grass lawn with mixed pine, birch, spruce trees; RO, DO, MO, HA |
| 8 Sept | 7 nn, 2 mm, 1 f/300 m^2^ (TJ); *Ir* | UP, Uppsala, Morkulleparken | 59°48'03"N, 17°38'33"E | Mixed broadleaved-coniferous woodland; RO, DO, HA |
| 21 Oct | 18 nn,1m, 2 ff/300 m^2^ (TJ); *Ir* | UP, Rådmansö, Djursnäs udde | 59°43'38"N, 18°55'44"E | Mixed woodland: *Corylus, Acer, Populus, Betula, Picea*; RO, MO, DO, HA |
| 21 Oct | 4 nn/300 m^2^ (TJ); *Ir* | UP, Rådmansö, Riddersholm | 59°43'22"N, 19°02'15"E | Cattle-grazed meadow with a few *Corylus* shrubs; CA, RO, MO, HA, DO |

^a^Include 30 or 60 stops; *Abbreviations*: *Ip*, *I. persulcatu*s; *Ir*, *I. ricinus,* n, nymph, nn, nymphs; m, adult male or meter; mm, adult males; f, adult female; ff, adult females; IF, KR, MB, EEP and TJ: initials of the collectors’ name (see foot-note below); 0, no tick collected; 1, one tick collected.

^b^*Abbreviations*: NB, Norrbotten (Middle boreal zone); VB, Västerbotten (Middle boreal zone); UP, Uppland (Boreo-nemoral zone).

^c^*Abbreviations*: CA, cattle (*Bos taurus*); DO, dog (*Canis lupus domesticus*); HA, varying hare (*Lepus timidus*); HO, horse (*Equus caballus*); MO, moose (*Alces alces*); RE, reindeer (*Rangifer tarandus*); RO, roe deer (*Capreolus capreolus*).

*Ticks were collected by IF, Isabella Fröjdman; KR, Kaj Rundgren; MB, Maria Brattsand; EEP, Erkki and Eine Partanen; and TJ, Thomas Jaenson.
