# Supplementary material for: First evidence of established populations of the taiga tick Ixodes persulcatus (Acari: Ixodidae) in Sweden
Source: Parasit Vectors. 2016 Jul 1;9:377. doi: 10.1186/s13071-016-1658-3 (PMC5116163; doi:10.1186/s13071-016-1658-3)
Supplement: Additionla file 2: Table S2. — Morphological characteristics distinguishing I. persulcatus from I. ricinus based on [3, 19]. (DOCX 22 kb) [file 13071_2016_1658_MOESM2_ESM.docx]

**Additional file 2: Table S2** Morphological characteristics distinguishing *Ixodes persulcatus* from *I. ricinus*, based on [[3](#_ENREF_3)] and [[19](#_ENREF_17)].

**Adult females:**

Auriculae reduced, indistinct. Internal spur on coxa I very long, sharply pointed, reaches to ~50 % of the width of posterior coxa..............................................................................*I. ricinus*

Auriculae distinct. Internal spur on coxa I shorter, reaches to only ~20 % of the width of posterior coxa..........................................................................................................*I. persulcatus*

**Adult males:**

Internal spur on coxa I very long, reaches to > 60 % of the width of posterior coxa….*I. ricinus*

Internal spur on coxa I shorter, reaches to < 40 % of the width of posterior coxa..*I. persulcatus*

**Nymphs:**

Setae on antero-lateral alloscutum 2.5× longer than setae on medial scutum.........*I. ricinus*

Setae on antero-lateral alloscutum only 1.3× longer than setae on medial scutum......................................................................................................................*I. persulcatus*
