# Supplementary figures and images for: First evidence of established populations of the taiga tick Ixodes persulcatus (Acari: Ixodidae) in Sweden
Source: Parasit Vectors. 2016 Jul 1;9:377. doi: 10.1186/s13071-016-1658-3 (PMC5116163; doi:10.1186/s13071-016-1658-3)

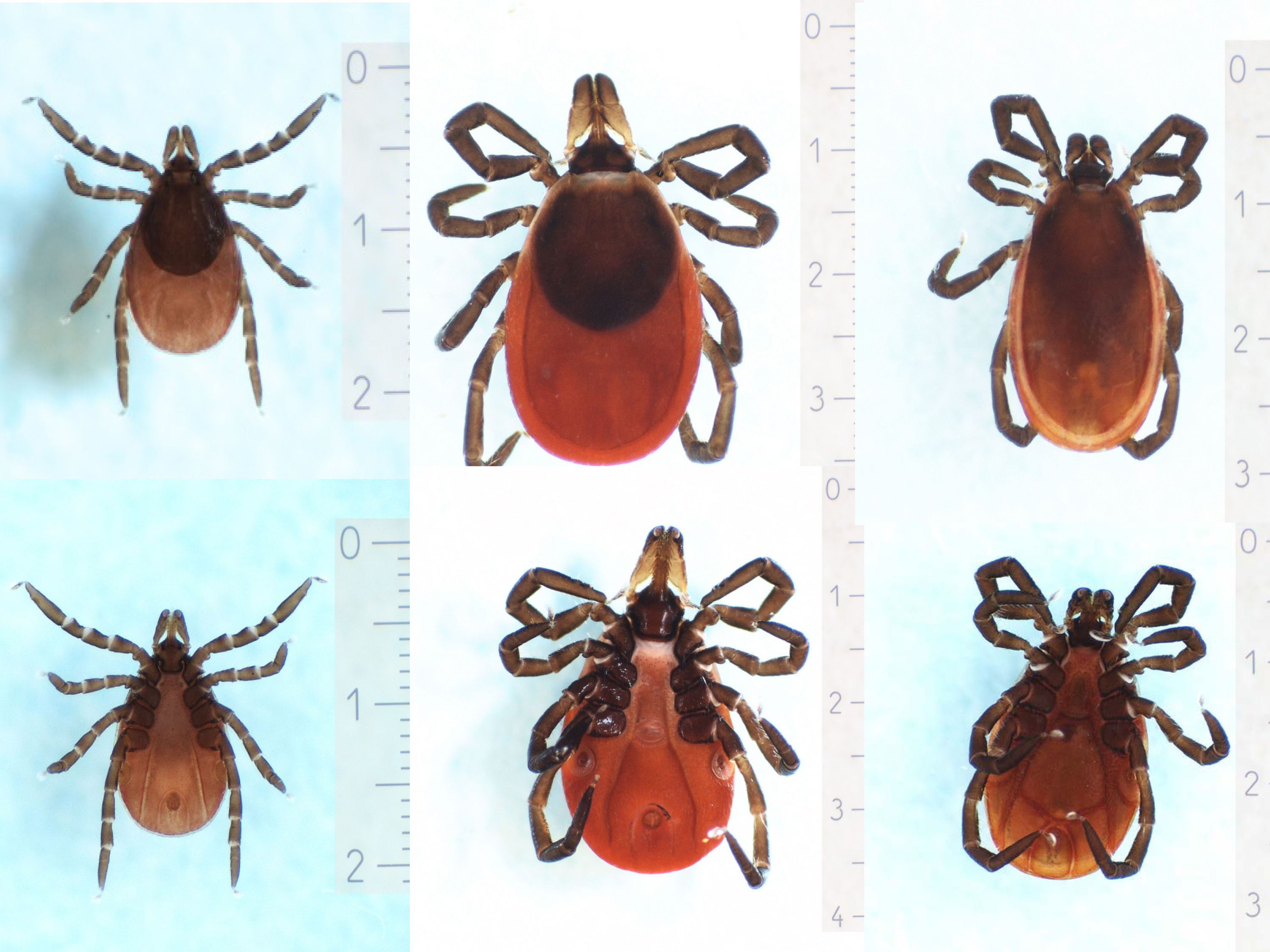

Supplement: Additional file 3: Figure S1. — Ixodes persulcatus collected on islands in the Bothnian Bay, northern Sweden in July 2015. Top row from left to right: dorsal view of nymph, adult female and adult male. Bottom row from left to right: ventral view of nymph, adult female and adult male. (TIF 32566 kb) [file 13071_2016_1658_MOESM3_ESM.tif]

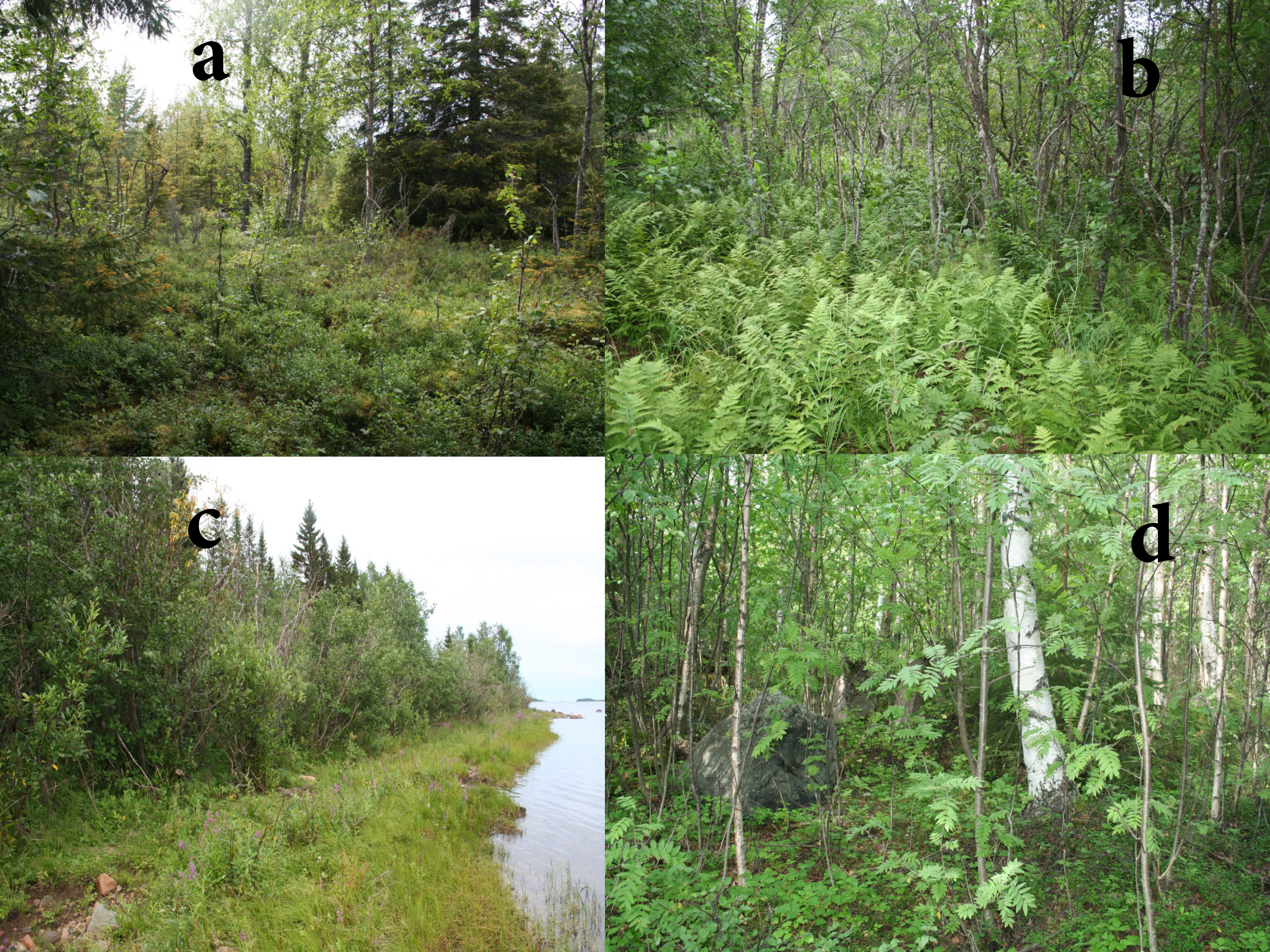

Supplement: Additional file 4: Figure S2. — Biotopes inhabited by the taiga tick, Ixodes persulcatus and moose, Alces alces in the Bothnian Bay archipelago, northern Sweden a. Mixed birch - spruce - rowan - willow and blueberry vegetation community on the island of Östra Knivskär, Haparanda municipality, 9th August 2015. b. Mixed birch - willow - grey alder woodland with field layer of bracken. Östra Knivskär, Haparanda municipality, 9th August 2015. c. Land upheaval mixed forest of grey alder, spruce, rowan, willow and Scots pine. Stora Hamnskär, Haparanda municipality, 9th August 2015. d. Mixed birch, spruce, rowan, pine and grey alder vegetation. Axelsvik, Kalix municipality, 11th August 2015. (TIF 38889 kb) [file 13071_2016_1658_MOESM4_ESM.tif]
